# Tris(μ_2_-methano­lato)-μ_3_-oxido-tris­{[(*E*)-4-chloro-2-({[2-(pyridin-2-yl)eth­yl]imino}­meth­yl)pheno­lato]manganese(III)} perchlorate–di­chloro­methane–diethyl ether (1/1.1/0.9)

**DOI:** 10.1107/S2056989017008982

**Published:** 2017-07-04

**Authors:** Rita Egekenze, Yilma Gultneh, Ray J. Butcher

**Affiliations:** a Department of Chemistry, Howard University, 525 College Street NW, Washington, DC 20059, USA

**Keywords:** crystal structure, trinuclear manganese complex, Jahn–Teller distortion, Schiff base complex

## Abstract

In the title compound, [Mn_3_(C_14_H_12_ClN_2_O)_3_(CH_3_O)_3_O]ClO_4_·1.1CH_2_Cl_2_·0.9C_4_H_10_O, the cation consists of a central Mn_3_O core with μ_2_-methano­late bridging between adjacent Mn^III^ atoms, thus giving each Mn^III^ atom a *mer*-O_3_ coordination environment. Six-coordination for each Mn^III^ atom is provided by the deprotonated Schiff base ligand (*E*)-4-chloro-2-({[2-(pyridin-2-yl)eth­yl]imino}­meth­yl)phenolate. There are extensive C—H⋯O and C—H⋯Cl inter­actions, which link the cations, anions, and solvent mol­ecules into a three-dimensional array.

## Abstract 2

The structure of a trinuclear manganese Schiff base complex with an Mn_3_O core is reported with the stoichiometry, C_45_H_42_Cl_3_Mn_3_N_6_O_7_, ClO_4_, C_4_H_10_O, CH_2_Cl_2_.

## Chemical context   

Single-mol­ecule magnets (SMMs) have attracted extensive attention because they are nanoscale magnetic particles of a well-defined size (Gatteschi & Sessoli 2003 ▸; Tasiopoulos *et al.*, 2004 ▸) and, in particular, manganese polynuclear manganese units have been investigated extensively in this respect. Employing salicylaldoxime ligands in manganese chemistry has proved to be extremely successful in the synthesis of new polynuclear complexes, including some SMMs (Milios *et al.*, 2004 ▸) and single-chain magnets (SCMs) (Feng *et al.*, 2009 ▸), suggesting that such ligands are excellent candidates for the preparation of polynuclear Mn complexes with inter­esting magnetic properties. A common motif in this chemistry is the formation of an Mn_3_O central core and a search of the Cambridge Structural Database (CSD; Groom *et al.*, 2016 ▸) for this moiety with each Mn atom surrounded by an additional N_2_O coordination environment gave over 500 hits. Most surprisingly in view of ubiquity of this type of ligand in transition metal coordination chemistry, there was not a single example in this list where the N_2_O coordination environment was supplied by a Schiff base ligand based on substituted salicyl­aldehyde derivatives. This paper reports the first example of such a structural type.

## Structural commentary   

**Figure 1:**
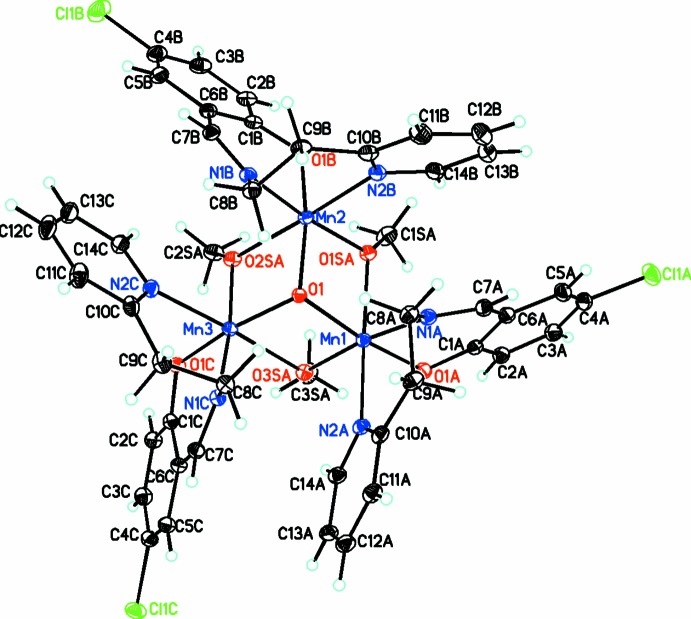
Diagram of the cation showing the atom labeling. Anions and solvent mol­ecules have been omitted for clarity. Atomic displacement parameters are drawn at the 30% probability level.

In the title compound, [Mn_3_(C_14_H_11_ClN_2_O)_3_(CH_3_O)_3_O]ClO_4_·1.1CH_2_Cl_2_·0.9C_4_H_10_O, the cation consists of a central Mn_3_O core with μ_2_-methano­late bridging between adjacent Mn^III^ atoms, thus giving each Mn^III^ atom a *mer*-O_3_ coordination environment (Fig. 1 ▸). Six-coordination for each Mn^III^ atom is provided by the deprotonated Schiff base ligand (*E*)-4-chloro-2-({[2-(pyridin-2-yl)eth­yl]imino}­meth­yl)phenolate, also coordinating in a *mer*-N_2_O fashion to each Mn^III^ atom. Thus the best description of the central Mn_3_O_4_ core, made up of the three Mn^III^ atoms, the central O and the bridging methano­late O atoms, is as a pseudo-cubane, missing one vertex. This can be seen by considering the Mn—O—Mn angles of 103.12 (6), 102.75 (6) and 101.75 (6)°.
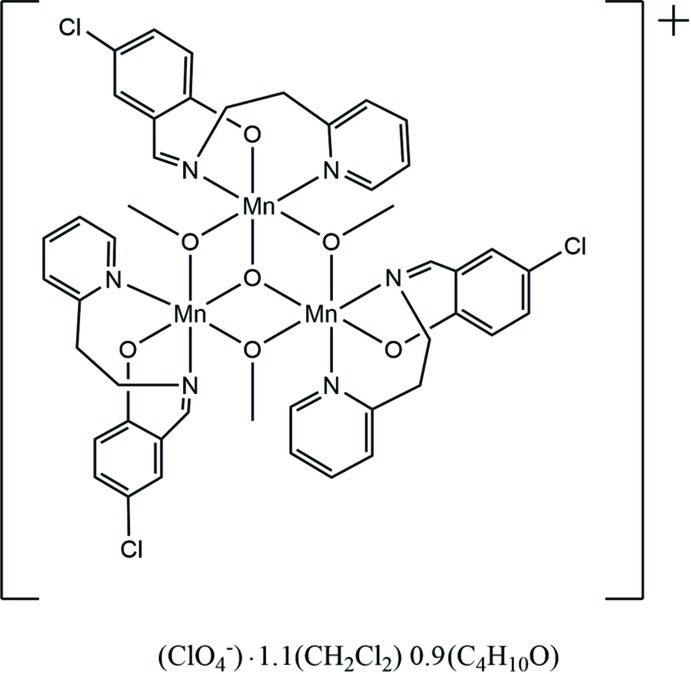


Since each Mn^III^ atom is in the +3 oxidation state and thus a high-spin *d^4^* ion, they are expected to exhibit Jahn–Teller distortion (Jahn & Teller, 1937 ▸). The most common type of Jahn–Teller distortion is a tetra­gonal distortion with the bond lengths along one *trans* axis being longer than expected. For each Mn^III^ atom, this is provided by the methano­late O and pyridine N atoms. Thus the Mn—O bond lengths involving the methano­late O atom are very asymmetric with one long (for the O atom involved in the Jahn–Teller distortion) and one short bond [2.1973 (14) and 1.8880 (14) Å; 2.2004 (13) and 1.8858 (13) Å; 2.2157 (14) and 1.8831 (13) Å]. The Mn—O bonds to the central O^2−^ are short [1.9427 (13), 1.9344 (13), 1.9429 (12) Å] as expected due to the respective charge of the two atoms.

For the coordinating Schiff base ligands, the Mn—O bond lengths are in the normal range for Mn^3+^ complexes [1.9020 (16), 1.8957 (14), and 1.8858 (13) Å] while the Mn—N bond lengths group into shorter Mn—N_amine_ [2.0202 (16), 2.0226 (16), and 2.0121 (16) Å] and longer Mn—N_py_ lengths [2.3640 (17), 2.4312 (16), and 2.3880 (17) Å].

## Supra­molecular features   

**Figure 2:**
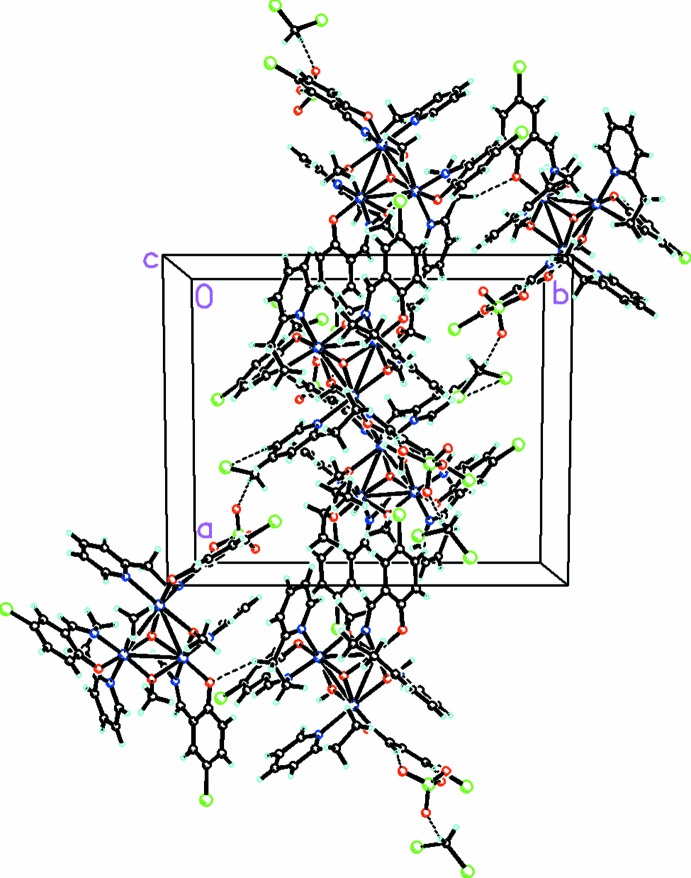
Packing diagram, viewed along the *b* axis, showing the extensive C—H⋯O and C—H⋯Cl inter­actions linking the cation, anion, and solvent mol­ecules into a three-dimensional array. For the disordered moieties, only the major disorder component is shown.

As seen in Fig. 2 ▸, there are extensive C—H⋯O and C—H⋯Cl inter­actions (Table 1 ▸), which link the cation anion and solvent mol­ecules into a three-dimensional array.

## Database survey   

A survey of the Cambridge Structural Database for Mn_3_O fragments where the Mn atoms are also coordinated by Schiff base ligands gave no hits. However, there were many instances of such units with salicylaldoxime ligands as this is a fertile field of research in the search for single mol­ecule magnets.

## Synthesis and crystallization   

A solution of the ligand C_14_H_13_ClNO (2.4793 g, 9.5 mmol) and an equivalent amount of tri­ethyl­amine (C_6_H_15_N; 1.3 ml, 9.5 mmol) both in methanol, was mixed with a methanol solution of Mn(ClO_4_)_2_ (1.7276 g, 4.8 mmol) in a 150 ml reaction flask. The mixture was refluxed for four h before it was cooled to room temperature. The solvent was reduced by rotary evaporation and the precipitate that formed was filtered by suction, washed with di­ethyl­ether and dried in a desiccator. Crystals suitable for X-ray diffraction were obtained by dissolving the compound in a mixture of methanol and di­chloro­methane and layering the solution with diethyl ether. The yield was 2.60 g (62%).

Characterization data for [C_50_H_54_Cl_6_Mn_3_N_6_O_12_] are as follows: IR (LiTaO_3_, KBr) (cm^−1^); 3073 (*w*), 2942 (*w*), 1616 (*m*), 1601 (*m*), 1567 (*w*), 1532 (*m*), 1485 (*w*), 1449 (*m*), 1437 (*w*), 1421 (*w*), 1449 (*m*), 1372 (*m*), 1280 (*s*), 1214 (*w*), 1188 (*m*), 1159 (*w*), 1080 (*s*), 1029 (*m*), 1012 (*w*), 970 (*w*), 960 (*w*), 917 (*w*), 872 (*w*), 862 (*w*), 846 (*m*), 808 (*m*), 760 (*m*), 781 (*s*), 760 (*m*), 706 (*s*), 662 (*m*). Uv–vis {λ_max_ (nm), (MeOH)}: 231 (21007.41), 285 (12846.85), 361 (4268.89), 421 (1690.93).

## Refinement   

Crystal data, data collection and structure refinement details are summarized in Table 2 ▸. The H atoms were positioned geometrically and allowed to ride on their parent atoms, with C—H = ranging from 0.93 to 0.98 Å and *U*_iso_(H) = *xU*_eq_(C), where *x* = 1.5 for methyl H atoms and 1.2 for all other C-bound H atoms. The di­chloro­methane and diethyl ether solvate mol­ecules were disordered. One of the di­chloro­methane solvate mol­ecules was disordered over three orientations with occupancies of 0.529 (3), 0.344 (3), and 0.127 (2) and was refined through the use of SAME and SIMU commands. The diethyl ether mol­ecule was disordered over two conformations and in addition there was a di­chloro­methane mol­ecule in the same vicinity. The diethyl ether mol­ecule was treated as being disordered and was refined with restraints to have similar metrical parameters using the SAME command. The occupancies of the two diethyl ether conformers [0.725 (3), 0.179 (3)], and the adjacent dicholormethane mol­ecule [0.0962 (18)] was summed to 1 through the use of the SUMP command. The displacement parameters of similar disordered species were restrained through the use of SIMU commands.

## Supplementary Material

Crystal structure: contains datablock(s) I. DOI: 10.1107/S2056989017008982/zl2705sup1.cif

Structure factors: contains datablock(s) I. DOI: 10.1107/S2056989017008982/zl2705Isup2.hkl

CCDC reference: 1556368

Additional supporting information:  crystallographic information; 3D view; checkCIF report

## Figures and Tables

**Table 1:** Hydrogen-bond geometry (Å, °)

*D*—H⋯*A*	*D*—H	H⋯*A*	*D*⋯*A*	*D*—H⋯*A*
C2*SA*—H2*SA*⋯O1*C*	0.98	2.42	2.991 (3)	117
C5*A*—H5*AA*⋯Cl8*S*	0.95	2.94	3.874 (11)	168
C5*A*—H5*AA*⋯Cl3*S* ^i^	0.95	2.72	3.39 (2)	128
C8*A*—H8*AA*⋯O1	0.99	2.40	3.061 (2)	123
C14*A*—H14*B*⋯O3*SA*	0.95	2.44	3.036 (2)	120
C3*B*—H3*BA*⋯Cl1*C* ^ii^	0.95	2.91	3.758 (2)	149
C8*B*—H8*BA*⋯O1	0.99	2.45	3.089 (2)	122
C9*B*—H9*BA*⋯O1*A* ^iii^	0.99	2.47	3.335 (2)	145
C9*B*—H9*BB*⋯O11^iv^	0.99	2.54	3.241 (3)	128
C14*B*—H14*A*⋯O1*SA*	0.95	2.53	3.116 (2)	120
C5*C*—H5*CA*⋯O14	0.95	2.38	3.300 (3)	164
C7*C*—H7*CA*⋯O13	0.95	2.55	3.478 (3)	164
C8*C*—H8*CA*⋯O1	0.99	2.43	3.079 (2)	123
C9*C*—H9*CA*⋯Cl7*S* ^v^	0.99	2.68	3.631 (11)	161
C9*C*—H9*CB*⋯O12^iv^	0.99	2.57	3.371 (3)	138
C9*C*—H9*CB*⋯O13^iv^	0.99	2.64	3.403 (3)	134
C13*C*—H13*C*⋯Cl1*S* ^vi^	0.95	2.80	3.698 (6)	159
C13*C*—H13*C*⋯Cl4*S* ^vi^	0.95	2.52	3.441 (12)	163
C13*C*—H13*C*⋯Cl5*S* ^vi^	0.95	2.91	3.843 (5)	169
C14*C*—H14*C*⋯O2*SA*	0.95	2.47	3.082 (3)	122
C1*DA*—H1*D*1⋯O11	0.99	2.53	3.201 (15)	125
C1*DA*—H1*D*1⋯O14	0.99	2.65	3.63 (2)	169
C1*DC*—H1*D*6⋯O11	0.99	2.24	3.176 (10)	158

Symmetry codes: (i) 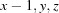; (ii) 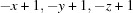; (iii) 
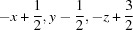
; (iv) 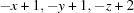; (v) 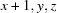; (vi) 
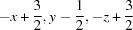
.

**Table 2:** Experimental details

Crystal data	
Chemical formula	[Mn_3_(C_14_H_12_ClN_2_O)_3_(CH_3_O)_3_O]ClO_4_·1.1CH_2_Cl_2_·0.9C_4_H_10_O
*M*_r_	1312.41
Crystal system, space group	Monoclinic, *P*2_1_/*n*
Temperature (K)	123
*a*, *b*, *c* (Å)	16.0002 (3), 19.3890 (2), 19.1631 (3)
β (°)	100.0727 (18)
*V* (Å^3^)	5853.31 (16)
*Z*	4
Radiation type	Mo *K*α
μ (mm^−1^)	0.98
Crystal size (mm)	0.32 × 0.27 × 0.18

Data collection	
Diffractometer	Agilent Xcalibur, Ruby, Gemini
Absorption correction	Gaussian (*CrysAlis PRO*; Agilent 2012 ▸)
*T*_min_, *T*_max_	0.757, 0.869
No. of measured, independent and observed [*I* > 2σ(*I*)] reflections	108163, 37613, 23139
*R*_int_	0.064
(sin θ/λ)_max_ (Å^−1^)	0.924

Refinement	
*R*[*F* ^2^ > 2σ(*F* ^2^)], *wR*(*F* ^2^), *S*	0.071, 0.166, 1.08
No. of reflections	37613
No. of parameters	833
No. of restraints	344
H-atom treatment	H-atom parameters constrained
Δρ_max_, Δρ_min_ (e Å^−3^)	0.74, −0.78

Computer programs: *CrysAlis PRO* (Agilent 2012 ▸), *SHELXS97* and *SHELXTL* (Sheldrick, 2008 ▸) and *SHELXL2016* (Sheldrick, 2015 ▸).

## supplementary crystallographic information

### Crystal data

**Table:**

[Mn_3_(C_14_H_12_ClN_2_O)_3_(CH_3_O)_3_O]ClO_4_·1.096CH_2_Cl_2_·0.906C_4_H_10_O	*F*(000) = 2688
*M**_r_* = 1312.41	*D*_x_ = 1.489 Mg m^−^^3^
Monoclinic, *P*2_1_/*n*	Mo *K*α radiation, λ = 0.71073 Å
*a* = 16.0002 (3) Å	Cell parameters from 18751 reflections
*b* = 19.3890 (2) Å	θ = 3.0–40.9°
*c* = 19.1631 (3) Å	µ = 0.98 mm^−^^1^
β = 100.0727 (18)°	*T* = 123 K
*V* = 5853.31 (16) Å^3^	Prism, brown-red
*Z* = 4	0.32 × 0.27 × 0.18 mm

### Data collection

**Table:**

Agilent Xcalibur, Ruby, Gemini diffractometer	23139 reflections with *I* > 2σ(*I*)
Detector resolution: 10.5081 pixels mm^-1^	*R*_int_ = 0.064
ω scans	θ_max_ = 41.0°, θ_min_ = 3.0°
Absorption correction: gaussian (CrysAlis PRO; Agilent 2012)	*h* = −26→29
*T*_min_ = 0.757, *T*_max_ = 0.869	*k* = −35→30
108163 measured reflections	*l* = −35→28
37613 independent reflections	

### Refinement

**Table:**

Refinement on *F*^2^	Secondary atom site location: difference Fourier map
Least-squares matrix: full	Hydrogen site location: inferred from neighbouring sites
*R*[*F*^2^ > 2σ(*F*^2^)] = 0.071	H-atom parameters constrained
*wR*(*F*^2^) = 0.166	*w* = 1/[σ^2^(*F*_o_^2^) + (0.051*P*)^2^ + 3.6302*P*] where *P* = (*F*_o_^2^ + 2*F*_c_^2^)/3
*S* = 1.08	(Δ/σ)_max_ = 0.002
37613 reflections	Δρ_max_ = 0.74 e Å^−^^3^
833 parameters	Δρ_min_ = −0.78 e Å^−^^3^
344 restraints	Extinction correction: SHELXL2016 (Sheldrick, 2015), Fc^*^=kFc[1+0.001xFc^2^λ^3^/sin(2θ)]^-1/4^
Primary atom site location: structure-invariant direct methods	Extinction coefficient: 0.00127 (15)

### Special details

**Table:**

Geometry. All esds (except the esd in the dihedral angle between two l.s. planes) are estimated using the full covariance matrix. The cell esds are taken into account individually in the estimation of esds in distances, angles and torsion angles; correlations between esds in cell parameters are only used when they are defined by crystal symmetry. An approximate (isotropic) treatment of cell esds is used for estimating esds involving l.s. planes.

### Fractional atomic coordinates and isotropic or equivalent isotropic displacement parameters (Å^2^)

**Table:**

	*x*	*y*	*z*	*U*_iso_*/*U*_eq_	Occ. (<1)
Mn1	0.24659 (2)	0.51964 (2)	0.71884 (2)	0.01770 (5)	
Mn2	0.26265 (2)	0.37124 (2)	0.67227 (2)	0.01828 (5)	
Mn3	0.41844 (2)	0.46209 (2)	0.70663 (2)	0.01751 (5)	
O1	0.31554 (8)	0.43753 (6)	0.74165 (7)	0.0173 (2)	
O1SA	0.18888 (9)	0.44462 (7)	0.63876 (7)	0.0214 (2)	
C1SA	0.14230 (16)	0.45174 (12)	0.56926 (11)	0.0326 (5)	
H1SA	0.091652	0.422457	0.563818	0.049*	
H1SB	0.177800	0.437713	0.534921	0.049*	
H1SC	0.125181	0.499986	0.560899	0.049*	
O2SA	0.37514 (9)	0.40444 (7)	0.62949 (7)	0.0215 (2)	
C2SA	0.39105 (15)	0.40900 (11)	0.55948 (11)	0.0287 (4)	
H2SA	0.423784	0.450877	0.554475	0.043*	
H2SB	0.337020	0.410770	0.526317	0.043*	
H2SC	0.423408	0.368510	0.549049	0.043*	
O3SA	0.33484 (9)	0.55173 (7)	0.67395 (7)	0.0209 (2)	
C3SA	0.32111 (16)	0.57908 (12)	0.60443 (11)	0.0301 (4)	
H3SA	0.282064	0.618314	0.601728	0.045*	
H3SB	0.296459	0.543417	0.570791	0.045*	
H3SC	0.375304	0.594451	0.592632	0.045*	
Cl1A	−0.19978 (4)	0.58318 (3)	0.61990 (4)	0.04071 (14)	
O1A	0.17184 (9)	0.59188 (7)	0.68086 (8)	0.0230 (3)	
N1A	0.16368 (11)	0.49440 (8)	0.78300 (9)	0.0208 (3)	
N2A	0.30287 (11)	0.58179 (8)	0.82259 (9)	0.0211 (3)	
C1A	0.08841 (12)	0.58770 (9)	0.66822 (11)	0.0220 (3)	
C2A	0.04248 (14)	0.62758 (10)	0.61312 (11)	0.0253 (4)	
H2AA	0.072256	0.656705	0.586003	0.030*	
C3A	−0.04500 (14)	0.62515 (11)	0.59776 (12)	0.0279 (4)	
H3AA	−0.074945	0.651635	0.559721	0.034*	
C4A	−0.08920 (14)	0.58376 (11)	0.63820 (13)	0.0293 (4)	
C5A	−0.04726 (14)	0.54533 (11)	0.69344 (13)	0.0291 (4)	
H5AA	−0.078507	0.518024	0.721041	0.035*	
C6A	0.04190 (13)	0.54624 (10)	0.70936 (11)	0.0240 (3)	
C7A	0.08429 (13)	0.50808 (10)	0.76994 (11)	0.0237 (3)	
H7AA	0.050597	0.491838	0.802712	0.028*	
C8A	0.20037 (13)	0.46199 (10)	0.85105 (10)	0.0224 (3)	
H8AA	0.248124	0.431758	0.844155	0.027*	
H8AB	0.156813	0.432956	0.867571	0.027*	
C9A	0.23208 (15)	0.51596 (10)	0.90712 (11)	0.0261 (4)	
H9AA	0.181842	0.539396	0.919813	0.031*	
H9AB	0.260696	0.491422	0.950013	0.031*	
C10A	0.29206 (13)	0.57106 (10)	0.88962 (10)	0.0220 (3)	
C11A	0.33388 (14)	0.61080 (11)	0.94515 (11)	0.0270 (4)	
H11A	0.326154	0.601517	0.992277	0.032*	
C12A	0.38669 (15)	0.66378 (11)	0.93212 (12)	0.0297 (4)	
H12A	0.416032	0.690924	0.969895	0.036*	
C13A	0.39596 (14)	0.67651 (10)	0.86257 (12)	0.0269 (4)	
H13A	0.430704	0.713239	0.851488	0.032*	
C14A	0.35361 (13)	0.63464 (10)	0.81018 (11)	0.0240 (3)	
H14B	0.360302	0.643238	0.762661	0.029*	
Cl1B	0.42659 (5)	0.09930 (4)	0.48119 (4)	0.04923 (18)	
O1B	0.22748 (10)	0.31485 (7)	0.59166 (8)	0.0240 (3)	
N1B	0.32701 (10)	0.29040 (8)	0.72229 (8)	0.0202 (3)	
N2B	0.16275 (11)	0.32735 (8)	0.74358 (9)	0.0234 (3)	
C1B	0.27593 (13)	0.26756 (9)	0.56916 (10)	0.0221 (3)	
C2B	0.26416 (15)	0.25324 (10)	0.49598 (11)	0.0269 (4)	
H2BA	0.223811	0.279141	0.464192	0.032*	
C3B	0.31036 (17)	0.20209 (11)	0.46962 (12)	0.0316 (5)	
H3BA	0.301113	0.192852	0.420182	0.038*	
C4B	0.37029 (16)	0.16419 (11)	0.51537 (13)	0.0320 (5)	
C5B	0.38412 (15)	0.17671 (11)	0.58709 (12)	0.0283 (4)	
H5BA	0.424838	0.150306	0.617999	0.034*	
C6B	0.33768 (13)	0.22892 (9)	0.61470 (11)	0.0228 (3)	
C7B	0.35206 (13)	0.23790 (9)	0.69073 (10)	0.0224 (3)	
H7BA	0.381992	0.202652	0.719195	0.027*	
C8B	0.33875 (13)	0.28906 (10)	0.80034 (10)	0.0219 (3)	
H8BA	0.345148	0.336795	0.818831	0.026*	
H8BB	0.391255	0.263376	0.819448	0.026*	
C9B	0.26346 (14)	0.25494 (10)	0.82502 (11)	0.0241 (3)	
H9BA	0.259888	0.206710	0.807786	0.029*	
H9BB	0.275234	0.253235	0.877460	0.029*	
C10B	0.17819 (14)	0.28793 (10)	0.80238 (11)	0.0248 (4)	
C11B	0.11494 (16)	0.27507 (12)	0.84253 (14)	0.0339 (5)	
H11B	0.127597	0.248372	0.884626	0.041*	
C12B	0.03395 (16)	0.30103 (13)	0.82129 (15)	0.0381 (5)	
H12C	−0.009105	0.293258	0.848814	0.046*	
C13B	0.01710 (15)	0.33870 (12)	0.75884 (15)	0.0354 (5)	
H13B	−0.038362	0.355625	0.741480	0.042*	
C14B	0.08288 (14)	0.35094 (11)	0.72260 (13)	0.0280 (4)	
H14A	0.071324	0.377540	0.680377	0.034*	
Cl1C	0.68540 (4)	0.76148 (3)	0.72233 (3)	0.03424 (12)	
O1C	0.50399 (9)	0.49956 (7)	0.66168 (7)	0.0223 (3)	
N1C	0.47280 (10)	0.50636 (8)	0.79836 (8)	0.0188 (3)	
N2C	0.50639 (11)	0.37137 (8)	0.76281 (9)	0.0229 (3)	
C1C	0.54086 (12)	0.55998 (9)	0.67642 (10)	0.0201 (3)	
C2C	0.57277 (14)	0.59545 (10)	0.62278 (11)	0.0247 (4)	
H2CA	0.564374	0.576653	0.576292	0.030*	
C3C	0.61601 (14)	0.65693 (11)	0.63604 (11)	0.0274 (4)	
H3CA	0.636613	0.680249	0.598870	0.033*	
C4C	0.62938 (13)	0.68475 (10)	0.70423 (12)	0.0256 (4)	
C5C	0.59863 (13)	0.65216 (10)	0.75820 (11)	0.0239 (3)	
H5CA	0.608288	0.671432	0.804491	0.029*	
C6C	0.55297 (12)	0.59041 (9)	0.74486 (10)	0.0203 (3)	
C7C	0.52521 (12)	0.55701 (10)	0.80406 (10)	0.0209 (3)	
H7CA	0.547140	0.573587	0.850292	0.025*	
C8C	0.46133 (13)	0.47129 (10)	0.86433 (9)	0.0216 (3)	
H8CA	0.405541	0.447796	0.857170	0.026*	
H8CB	0.462536	0.505630	0.902726	0.026*	
C9C	0.53155 (13)	0.41895 (11)	0.88516 (11)	0.0261 (4)	
H9CA	0.585857	0.444338	0.897073	0.031*	
H9CB	0.521646	0.395597	0.928930	0.031*	
C10C	0.54219 (12)	0.36411 (10)	0.83156 (11)	0.0242 (3)	
C11C	0.59328 (15)	0.30743 (12)	0.85498 (13)	0.0330 (5)	
H11C	0.616876	0.302793	0.903809	0.040*	
C12C	0.60943 (16)	0.25828 (12)	0.80726 (15)	0.0366 (5)	
H12B	0.643634	0.219271	0.822715	0.044*	
C13C	0.57475 (15)	0.26673 (12)	0.73594 (14)	0.0335 (5)	
H13C	0.585880	0.234393	0.701464	0.040*	
C14C	0.52358 (14)	0.32358 (11)	0.71666 (12)	0.0274 (4)	
H14C	0.499295	0.329068	0.668079	0.033*	
Cl1	0.62722 (4)	0.65431 (3)	0.99642 (3)	0.03190 (11)	
O11	0.71139 (12)	0.64631 (11)	1.03643 (10)	0.0418 (4)	
O12	0.57862 (13)	0.69681 (11)	1.03593 (10)	0.0445 (4)	
O13	0.58792 (15)	0.58751 (11)	0.98449 (11)	0.0495 (5)	
O14	0.63084 (17)	0.68626 (12)	0.92951 (10)	0.0554 (6)	
O1S	−0.1958 (2)	0.4106 (2)	0.6690 (3)	0.0658 (9)	0.725 (3)
C1S	−0.1231 (4)	0.3811 (3)	0.5773 (3)	0.0745 (15)	0.725 (3)
H1S1	−0.094270	0.425652	0.586542	0.112*	0.725 (3)
H1S2	−0.129630	0.370303	0.526670	0.112*	0.725 (3)
H1S3	−0.089252	0.345058	0.604814	0.112*	0.725 (3)
C2S	−0.2026 (4)	0.3845 (4)	0.5969 (4)	0.0827 (14)	0.725 (3)
H2S1	−0.228517	0.337982	0.593833	0.099*	0.725 (3)
H2S2	−0.239984	0.415217	0.563894	0.099*	0.725 (3)
C3S	−0.2751 (3)	0.4180 (3)	0.6887 (4)	0.0759 (12)	0.725 (3)
H3S1	−0.303259	0.372531	0.688295	0.091*	0.725 (3)
H3S2	−0.311363	0.448581	0.654740	0.091*	0.725 (3)
C4S	−0.2637 (5)	0.4483 (4)	0.7613 (5)	0.0880 (17)	0.725 (3)
H4S1	−0.229174	0.417182	0.794929	0.132*	0.725 (3)
H4S2	−0.319360	0.454606	0.775159	0.132*	0.725 (3)
H4S3	−0.235177	0.493049	0.761533	0.132*	0.725 (3)
O1SB	−0.2100 (11)	0.4153 (10)	0.6983 (10)	0.0729 (16)	0.179 (3)
C1SB	−0.3235 (13)	0.3982 (13)	0.5931 (14)	0.079 (3)	0.179 (3)
H1S4	−0.331209	0.378422	0.545306	0.118*	0.179 (3)
H1S5	−0.360683	0.374317	0.620813	0.118*	0.179 (3)
H1S6	−0.337924	0.447326	0.589970	0.118*	0.179 (3)
C2SB	−0.2390 (13)	0.3902 (12)	0.6261 (12)	0.0753 (17)	0.179 (3)
H2S3	−0.203415	0.412828	0.595387	0.090*	0.179 (3)
H2S4	−0.226021	0.340312	0.626023	0.090*	0.179 (3)
C3SB	−0.2748 (15)	0.4335 (16)	0.7331 (14)	0.0732 (17)	0.179 (3)
H3S3	−0.312747	0.393683	0.736257	0.088*	0.179 (3)
H3S4	−0.308753	0.471613	0.708069	0.088*	0.179 (3)
C4SB	−0.2316 (16)	0.4564 (14)	0.8070 (13)	0.078 (3)	0.179 (3)
H4S4	−0.274789	0.470141	0.834748	0.116*	0.179 (3)
H4S5	−0.198091	0.418126	0.830822	0.116*	0.179 (3)
H4S6	−0.194120	0.495604	0.802797	0.116*	0.179 (3)
C1DD	−0.270 (2)	0.4917 (18)	0.7921 (12)	0.078 (2)	0.0962 (18)
H1D7	−0.327480	0.478012	0.769269	0.094*	0.0962 (18)
H1D8	−0.255062	0.535095	0.769690	0.094*	0.0962 (18)
Cl7S	−0.2679 (7)	0.5064 (6)	0.8846 (6)	0.079 (3)	0.0962 (18)
Cl8S	−0.1969 (8)	0.4266 (6)	0.7779 (8)	0.084 (2)	0.0962 (18)
C1DA	0.8539 (13)	0.6470 (9)	0.9382 (10)	0.116 (3)	0.344 (3)
H1D1	0.791517	0.650680	0.933815	0.139*	0.344 (3)
H1D2	0.874914	0.616727	0.979130	0.139*	0.344 (3)
Cl1S	0.8797 (4)	0.6074 (3)	0.8566 (4)	0.1136 (18)	0.344 (3)
Cl2S	0.9009 (6)	0.7322 (4)	0.9545 (5)	0.104 (2)	0.344 (3)
C1DB	0.842 (3)	0.5960 (15)	0.918 (3)	0.116 (4)	0.127 (2)
H1D3	0.857139	0.574021	0.964774	0.139*	0.127 (2)
H1D4	0.780027	0.601295	0.905291	0.139*	0.127 (2)
Cl3S	0.8840 (15)	0.5479 (10)	0.8509 (12)	0.179 (5)	0.127 (2)
Cl4S	0.8973 (8)	0.6795 (6)	0.9146 (7)	0.100 (3)	0.127 (2)
C1DC	0.8304 (8)	0.7012 (6)	0.9321 (7)	0.117 (3)	0.529 (3)
H1D5	0.785326	0.727969	0.901941	0.141*	0.529 (3)
H1D6	0.807464	0.685353	0.974120	0.141*	0.529 (3)
Cl5S	0.8540 (3)	0.6277 (2)	0.8840 (3)	0.1189 (12)	0.529 (3)
Cl6S	0.9171 (3)	0.7564 (2)	0.9609 (2)	0.0799 (12)	0.529 (3)

### Atomic displacement parameters (Å^2^)

**Table:**

	*U*^11^	*U*^22^	*U*^33^	*U*^12^	*U*^13^	*U*^23^
Mn1	0.01926 (12)	0.01531 (10)	0.01826 (11)	0.00037 (9)	0.00256 (9)	0.00121 (9)
Mn2	0.02095 (12)	0.01474 (10)	0.01806 (11)	−0.00015 (9)	0.00038 (9)	−0.00042 (9)
Mn3	0.01911 (12)	0.01785 (10)	0.01538 (10)	−0.00121 (9)	0.00254 (9)	−0.00030 (9)
O1	0.0177 (5)	0.0165 (5)	0.0172 (5)	0.0001 (4)	0.0018 (4)	0.0002 (4)
O1SA	0.0224 (6)	0.0199 (5)	0.0197 (5)	0.0008 (5)	−0.0022 (5)	0.0000 (5)
C1SA	0.0384 (12)	0.0311 (10)	0.0235 (9)	0.0090 (9)	−0.0075 (8)	−0.0013 (8)
O2SA	0.0274 (7)	0.0220 (6)	0.0156 (5)	−0.0037 (5)	0.0049 (5)	−0.0017 (5)
C2SA	0.0362 (11)	0.0304 (9)	0.0206 (8)	−0.0079 (8)	0.0084 (8)	−0.0042 (7)
O3SA	0.0237 (6)	0.0205 (5)	0.0182 (5)	−0.0010 (5)	0.0028 (5)	0.0036 (5)
C3SA	0.0367 (11)	0.0316 (10)	0.0226 (8)	0.0079 (9)	0.0071 (8)	0.0096 (8)
Cl1A	0.0217 (2)	0.0395 (3)	0.0579 (4)	0.0017 (2)	−0.0015 (2)	0.0102 (3)
O1A	0.0204 (6)	0.0190 (5)	0.0288 (7)	0.0007 (5)	0.0020 (5)	0.0037 (5)
N1A	0.0227 (7)	0.0175 (6)	0.0219 (7)	0.0008 (5)	0.0031 (6)	0.0012 (5)
N2A	0.0245 (7)	0.0174 (6)	0.0204 (6)	0.0025 (5)	0.0009 (6)	−0.0003 (5)
C1A	0.0223 (8)	0.0167 (7)	0.0268 (8)	0.0010 (6)	0.0034 (7)	−0.0007 (6)
C2A	0.0266 (9)	0.0217 (8)	0.0268 (9)	0.0018 (7)	0.0029 (7)	0.0024 (7)
C3A	0.0258 (9)	0.0257 (9)	0.0299 (9)	0.0036 (7)	−0.0020 (7)	0.0009 (8)
C4A	0.0217 (9)	0.0265 (9)	0.0380 (11)	0.0024 (7)	0.0003 (8)	0.0000 (8)
C5A	0.0223 (9)	0.0261 (9)	0.0384 (11)	0.0006 (7)	0.0037 (8)	0.0033 (8)
C6A	0.0233 (8)	0.0185 (7)	0.0297 (9)	0.0012 (6)	0.0035 (7)	0.0019 (7)
C7A	0.0235 (8)	0.0208 (7)	0.0273 (9)	0.0007 (6)	0.0063 (7)	0.0019 (7)
C8A	0.0276 (9)	0.0196 (7)	0.0202 (7)	0.0020 (7)	0.0049 (7)	0.0033 (6)
C9A	0.0341 (10)	0.0234 (8)	0.0211 (8)	0.0021 (8)	0.0060 (7)	−0.0002 (7)
C10A	0.0246 (8)	0.0199 (7)	0.0207 (7)	0.0061 (6)	0.0014 (6)	−0.0007 (6)
C11A	0.0293 (10)	0.0280 (9)	0.0219 (8)	0.0063 (8)	−0.0005 (7)	−0.0043 (7)
C12A	0.0295 (10)	0.0278 (9)	0.0288 (9)	0.0043 (8)	−0.0030 (8)	−0.0095 (8)
C13A	0.0266 (9)	0.0198 (8)	0.0320 (10)	0.0013 (7)	−0.0007 (8)	−0.0045 (7)
C14A	0.0269 (9)	0.0191 (7)	0.0250 (8)	0.0018 (7)	0.0014 (7)	0.0006 (7)
Cl1B	0.0617 (4)	0.0414 (3)	0.0512 (4)	0.0062 (3)	0.0280 (4)	−0.0137 (3)
O1B	0.0281 (7)	0.0185 (5)	0.0234 (6)	−0.0013 (5)	−0.0012 (5)	−0.0027 (5)
N1B	0.0222 (7)	0.0176 (6)	0.0200 (6)	−0.0006 (5)	0.0018 (5)	0.0014 (5)
N2B	0.0243 (7)	0.0191 (6)	0.0265 (7)	−0.0020 (6)	0.0037 (6)	−0.0003 (6)
C1B	0.0269 (9)	0.0175 (7)	0.0219 (8)	−0.0063 (6)	0.0039 (7)	−0.0021 (6)
C2B	0.0365 (11)	0.0214 (8)	0.0224 (8)	−0.0074 (7)	0.0044 (8)	−0.0017 (7)
C3B	0.0457 (13)	0.0262 (9)	0.0253 (9)	−0.0094 (9)	0.0129 (9)	−0.0043 (8)
C4B	0.0395 (12)	0.0257 (9)	0.0353 (11)	−0.0045 (8)	0.0188 (10)	−0.0077 (8)
C5B	0.0306 (10)	0.0227 (8)	0.0327 (10)	0.0003 (7)	0.0092 (8)	−0.0035 (8)
C6B	0.0254 (9)	0.0185 (7)	0.0250 (8)	−0.0028 (6)	0.0056 (7)	−0.0014 (6)
C7B	0.0243 (8)	0.0182 (7)	0.0239 (8)	0.0004 (6)	0.0023 (7)	0.0013 (6)
C8B	0.0255 (8)	0.0193 (7)	0.0198 (7)	0.0003 (6)	0.0008 (6)	0.0017 (6)
C9B	0.0311 (10)	0.0184 (7)	0.0226 (8)	−0.0021 (7)	0.0044 (7)	0.0019 (6)
C10B	0.0292 (9)	0.0189 (7)	0.0263 (9)	−0.0034 (7)	0.0052 (7)	−0.0021 (7)
C11B	0.0362 (12)	0.0300 (10)	0.0378 (12)	−0.0049 (9)	0.0128 (10)	0.0046 (9)
C12B	0.0312 (11)	0.0373 (11)	0.0490 (14)	−0.0063 (10)	0.0161 (11)	0.0022 (11)
C13B	0.0253 (10)	0.0313 (10)	0.0505 (14)	−0.0022 (8)	0.0096 (10)	0.0001 (10)
C14B	0.0260 (9)	0.0228 (8)	0.0345 (10)	−0.0023 (7)	0.0035 (8)	0.0012 (8)
Cl1C	0.0350 (3)	0.0292 (2)	0.0401 (3)	−0.0133 (2)	0.0110 (2)	−0.0032 (2)
O1C	0.0257 (7)	0.0222 (6)	0.0197 (6)	−0.0041 (5)	0.0057 (5)	−0.0023 (5)
N1C	0.0194 (6)	0.0201 (6)	0.0168 (6)	0.0002 (5)	0.0028 (5)	0.0014 (5)
N2C	0.0207 (7)	0.0218 (7)	0.0263 (7)	0.0016 (6)	0.0043 (6)	0.0031 (6)
C1C	0.0197 (7)	0.0207 (7)	0.0198 (7)	0.0001 (6)	0.0034 (6)	0.0006 (6)
C2C	0.0282 (9)	0.0265 (8)	0.0212 (8)	−0.0032 (7)	0.0089 (7)	−0.0006 (7)
C3C	0.0292 (10)	0.0281 (9)	0.0268 (9)	−0.0037 (8)	0.0100 (8)	0.0019 (8)
C4C	0.0243 (9)	0.0229 (8)	0.0303 (9)	−0.0040 (7)	0.0074 (8)	−0.0006 (7)
C5C	0.0249 (9)	0.0241 (8)	0.0232 (8)	−0.0042 (7)	0.0052 (7)	−0.0026 (7)
C6C	0.0212 (8)	0.0197 (7)	0.0198 (7)	−0.0020 (6)	0.0031 (6)	−0.0003 (6)
C7C	0.0219 (8)	0.0223 (7)	0.0179 (7)	−0.0010 (6)	0.0019 (6)	−0.0013 (6)
C8C	0.0238 (8)	0.0244 (8)	0.0165 (7)	−0.0005 (6)	0.0030 (6)	0.0022 (6)
C9C	0.0242 (9)	0.0321 (9)	0.0210 (8)	0.0014 (7)	0.0012 (7)	0.0057 (7)
C10C	0.0187 (8)	0.0264 (8)	0.0276 (9)	0.0011 (7)	0.0045 (7)	0.0085 (7)
C11C	0.0288 (10)	0.0354 (11)	0.0350 (11)	0.0085 (9)	0.0063 (9)	0.0139 (9)
C12C	0.0304 (11)	0.0304 (10)	0.0509 (14)	0.0110 (9)	0.0125 (10)	0.0142 (10)
C13C	0.0291 (10)	0.0271 (9)	0.0465 (13)	0.0054 (8)	0.0126 (10)	0.0039 (9)
C14C	0.0261 (9)	0.0249 (8)	0.0318 (10)	0.0012 (7)	0.0071 (8)	0.0003 (8)
Cl1	0.0348 (3)	0.0391 (3)	0.0204 (2)	−0.0060 (2)	0.00105 (19)	−0.00104 (19)
O11	0.0373 (9)	0.0468 (10)	0.0370 (9)	0.0018 (8)	−0.0055 (7)	−0.0042 (8)
O12	0.0397 (10)	0.0540 (11)	0.0388 (10)	0.0000 (9)	0.0044 (8)	−0.0111 (9)
O13	0.0572 (13)	0.0466 (11)	0.0461 (11)	−0.0201 (10)	0.0130 (10)	−0.0131 (9)
O14	0.0770 (16)	0.0629 (14)	0.0253 (8)	−0.0066 (12)	0.0065 (10)	0.0108 (9)
O1S	0.0426 (16)	0.0521 (16)	0.104 (3)	−0.0025 (13)	0.0178 (17)	0.0312 (19)
C1S	0.091 (4)	0.074 (3)	0.052 (3)	0.000 (3)	−0.008 (3)	0.001 (3)
C2S	0.069 (3)	0.074 (3)	0.097 (3)	−0.010 (2)	−0.007 (3)	0.017 (3)
C3S	0.050 (2)	0.061 (2)	0.120 (3)	−0.0022 (18)	0.024 (2)	0.032 (2)
C4S	0.066 (3)	0.075 (3)	0.132 (4)	0.010 (3)	0.040 (3)	0.035 (3)
O1SB	0.049 (3)	0.056 (3)	0.115 (3)	−0.002 (3)	0.017 (3)	0.031 (3)
C1SB	0.069 (6)	0.067 (5)	0.101 (6)	−0.003 (5)	0.015 (5)	0.024 (6)
C2SB	0.055 (3)	0.062 (3)	0.109 (3)	−0.004 (3)	0.012 (3)	0.027 (3)
C3SB	0.050 (3)	0.058 (3)	0.114 (4)	−0.001 (3)	0.023 (3)	0.031 (3)
C4SB	0.053 (5)	0.069 (5)	0.117 (5)	0.011 (4)	0.034 (5)	0.026 (5)
C1DD	0.058 (4)	0.065 (4)	0.117 (5)	0.006 (4)	0.029 (4)	0.030 (4)
Cl7S	0.066 (5)	0.087 (5)	0.098 (5)	0.017 (4)	0.054 (4)	0.041 (5)
Cl8S	0.064 (4)	0.064 (4)	0.127 (5)	0.009 (3)	0.025 (4)	0.027 (4)
C1DA	0.107 (6)	0.127 (7)	0.127 (6)	−0.012 (6)	0.059 (6)	0.021 (6)
Cl1S	0.116 (4)	0.101 (4)	0.137 (4)	−0.024 (3)	0.058 (3)	0.021 (3)
Cl2S	0.107 (5)	0.130 (6)	0.072 (3)	0.051 (4)	0.008 (3)	−0.017 (4)
C1DB	0.121 (8)	0.115 (8)	0.127 (8)	−0.013 (8)	0.063 (7)	0.013 (8)
Cl3S	0.191 (10)	0.167 (10)	0.186 (10)	−0.015 (10)	0.054 (9)	0.014 (10)
Cl4S	0.102 (6)	0.099 (6)	0.107 (6)	−0.013 (5)	0.045 (5)	−0.007 (5)
C1DC	0.093 (5)	0.132 (7)	0.141 (6)	−0.015 (5)	0.060 (5)	0.004 (6)
Cl5S	0.136 (3)	0.085 (2)	0.144 (4)	−0.036 (2)	0.047 (3)	0.001 (2)
Cl6S	0.0647 (15)	0.113 (3)	0.069 (2)	0.0062 (17)	0.0313 (15)	0.029 (2)

### Geometric parameters (Å, º)

**Table:**

Mn1—O3SA	1.8831 (13)	C10B—C11B	1.397 (3)
Mn1—O1A	1.9020 (14)	C11B—C12B	1.383 (4)
Mn1—O1	1.9427 (13)	C11B—H11B	0.9500
Mn1—N1A	2.0202 (16)	C12B—C13B	1.388 (4)
Mn1—O1SA	2.1973 (14)	C12B—H12C	0.9500
Mn1—N2A	2.3640 (17)	C13B—C14B	1.379 (3)
Mn1—Mn3	3.0143 (4)	C13B—H13B	0.9500
Mn1—Mn2	3.0368 (4)	C14B—H14A	0.9500
Mn2—O1SA	1.8880 (14)	Cl1C—C4C	1.740 (2)
Mn2—O1B	1.8957 (14)	O1C—C1C	1.320 (2)
Mn2—O1	1.9344 (13)	N1C—C7C	1.284 (2)
Mn2—N1B	2.0226 (16)	N1C—C8C	1.475 (2)
Mn2—O2SA	2.2004 (13)	N2C—C14C	1.342 (3)
Mn2—N2B	2.4312 (16)	N2C—C10C	1.349 (3)
Mn2—Mn3	3.0292 (4)	C1C—C2C	1.405 (3)
Mn3—O1C	1.8858 (13)	C1C—C6C	1.420 (3)
Mn3—O2SA	1.8858 (14)	C2C—C3C	1.379 (3)
Mn3—O1	1.9429 (12)	C2C—H2CA	0.9500
Mn3—N1C	2.0121 (16)	C3C—C4C	1.395 (3)
Mn3—O3SA	2.2157 (14)	C3C—H3CA	0.9500
Mn3—N2C	2.3880 (17)	C4C—C5C	1.374 (3)
O1SA—C1SA	1.415 (3)	C5C—C6C	1.403 (3)
C1SA—H1SA	0.9800	C5C—H5CA	0.9500
C1SA—H1SB	0.9800	C6C—C7C	1.442 (3)
C1SA—H1SC	0.9800	C7C—H7CA	0.9500
O2SA—C2SA	1.411 (2)	C8C—C9C	1.515 (3)
C2SA—H2SA	0.9800	C8C—H8CA	0.9900
C2SA—H2SB	0.9800	C8C—H8CB	0.9900
C2SA—H2SC	0.9800	C9C—C10C	1.509 (3)
O3SA—C3SA	1.415 (2)	C9C—H9CA	0.9900
C3SA—H3SA	0.9800	C9C—H9CB	0.9900
C3SA—H3SB	0.9800	C10C—C11C	1.396 (3)
C3SA—H3SC	0.9800	C11C—C12C	1.376 (4)
Cl1A—C4A	1.743 (2)	C11C—H11C	0.9500
O1A—C1A	1.317 (2)	C12C—C13C	1.392 (4)
N1A—C7A	1.279 (3)	C12C—H12B	0.9500
N1A—C8A	1.474 (2)	C13C—C14C	1.384 (3)
N2A—C10A	1.342 (2)	C13C—H13C	0.9500
N2A—C14A	1.354 (3)	C14C—H14C	0.9500
C1A—C2A	1.408 (3)	Cl1—O14	1.4342 (19)
C1A—C6A	1.424 (3)	Cl1—O12	1.436 (2)
C2A—C3A	1.380 (3)	Cl1—O11	1.437 (2)
C2A—H2AA	0.9500	Cl1—O13	1.440 (2)
C3A—C4A	1.391 (3)	O1S—C3S	1.393 (7)
C3A—H3AA	0.9500	O1S—C2S	1.456 (8)
C4A—C5A	1.370 (3)	C1S—C2S	1.391 (9)
C5A—C6A	1.406 (3)	C1S—H1S1	0.9800
C5A—H5AA	0.9500	C1S—H1S2	0.9800
C6A—C7A	1.442 (3)	C1S—H1S3	0.9800
C7A—H7AA	0.9500	C2S—H2S1	0.9900
C8A—C9A	1.522 (3)	C2S—H2S2	0.9900
C8A—H8AA	0.9900	C3S—C4S	1.491 (10)
C8A—H8AB	0.9900	C3S—H3S1	0.9900
C9A—C10A	1.512 (3)	C3S—H3S2	0.9900
C9A—H9AA	0.9900	C4S—H4S1	0.9800
C9A—H9AB	0.9900	C4S—H4S2	0.9800
C10A—C11A	1.387 (3)	C4S—H4S3	0.9800
C11A—C12A	1.380 (3)	O1SB—C3SB	1.374 (18)
C11A—H11A	0.9500	O1SB—C2SB	1.464 (18)
C12A—C13A	1.389 (3)	C1SB—C2SB	1.397 (18)
C12A—H12A	0.9500	C1SB—H1S4	0.9800
C13A—C14A	1.374 (3)	C1SB—H1S5	0.9800
C13A—H13A	0.9500	C1SB—H1S6	0.9800
C14A—H14B	0.9500	C2SB—H2S3	0.9900
Cl1B—C4B	1.741 (2)	C2SB—H2S4	0.9900
O1B—C1B	1.321 (2)	C3SB—C4SB	1.53 (2)
N1B—C7B	1.284 (2)	C3SB—H3S3	0.9900
N1B—C8B	1.475 (2)	C3SB—H3S4	0.9900
N2B—C10B	1.348 (3)	C4SB—H4S4	0.9800
N2B—C14B	1.351 (3)	C4SB—H4S5	0.9800
C1B—C2B	1.410 (3)	C4SB—H4S6	0.9800
C1B—C6B	1.413 (3)	C1DD—Cl8S	1.769 (19)
C2B—C3B	1.384 (3)	C1DD—Cl7S	1.79 (2)
C2B—H2BA	0.9500	C1DD—H1D7	0.9900
C3B—C4B	1.391 (4)	C1DD—H1D8	0.9900
C3B—H3BA	0.9500	C1DA—Cl2S	1.820 (15)
C4B—C5B	1.375 (3)	C1DA—Cl1S	1.851 (14)
C5B—C6B	1.412 (3)	C1DA—H1D1	0.9900
C5B—H5BA	0.9500	C1DA—H1D2	0.9900
C6B—C7B	1.445 (3)	C1DB—Cl3S	1.802 (19)
C7B—H7BA	0.9500	C1DB—Cl4S	1.847 (19)
C8B—C9B	1.521 (3)	C1DB—H1D3	0.9900
C8B—H8BA	0.9900	C1DB—H1D4	0.9900
C8B—H8BB	0.9900	C1DC—Cl6S	1.763 (11)
C9B—C10B	1.501 (3)	C1DC—Cl5S	1.774 (11)
C9B—H9BA	0.9900	C1DC—H1D5	0.9900
C9B—H9BB	0.9900	C1DC—H1D6	0.9900
			
O3SA—Mn1—O1A	93.06 (6)	C3B—C2B—H2BA	119.4
O3SA—Mn1—O1	86.09 (6)	C1B—C2B—H2BA	119.4
O1A—Mn1—O1	169.71 (6)	C2B—C3B—C4B	120.1 (2)
O3SA—Mn1—N1A	169.34 (7)	C2B—C3B—H3BA	119.9
O1A—Mn1—N1A	88.59 (6)	C4B—C3B—H3BA	119.9
O1—Mn1—N1A	94.13 (6)	C5B—C4B—C3B	120.6 (2)
O3SA—Mn1—O1SA	99.02 (6)	C5B—C4B—Cl1B	120.0 (2)
O1A—Mn1—O1SA	93.49 (6)	C3B—C4B—Cl1B	119.33 (17)
O1—Mn1—O1SA	76.54 (5)	C4B—C5B—C6B	119.9 (2)
N1A—Mn1—O1SA	91.39 (6)	C4B—C5B—H5BA	120.1
O3SA—Mn1—N2A	90.57 (6)	C6B—C5B—H5BA	120.1
O1A—Mn1—N2A	93.90 (6)	C5B—C6B—C1B	120.35 (19)
O1—Mn1—N2A	96.36 (6)	C5B—C6B—C7B	117.56 (19)
N1A—Mn1—N2A	78.80 (6)	C1B—C6B—C7B	121.98 (17)
O1SA—Mn1—N2A	167.55 (5)	N1B—C7B—C6B	124.25 (18)
O3SA—Mn1—Mn3	47.14 (4)	N1B—C7B—H7BA	117.9
O1A—Mn1—Mn3	139.82 (4)	C6B—C7B—H7BA	117.9
O1—Mn1—Mn3	39.13 (4)	N1B—C8B—C9B	110.84 (16)
N1A—Mn1—Mn3	131.49 (5)	N1B—C8B—H8BA	109.5
O1SA—Mn1—Mn3	88.76 (4)	C9B—C8B—H8BA	109.5
N2A—Mn1—Mn3	92.03 (4)	N1B—C8B—H8BB	109.5
O3SA—Mn1—Mn2	94.02 (4)	C9B—C8B—H8BB	109.5
O1A—Mn1—Mn2	131.69 (5)	H8BA—C8B—H8BB	108.1
O1—Mn1—Mn2	38.34 (4)	C10B—C9B—C8B	117.03 (16)
N1A—Mn1—Mn2	92.73 (5)	C10B—C9B—H9BA	108.0
O1SA—Mn1—Mn2	38.21 (4)	C8B—C9B—H9BA	108.0
N2A—Mn1—Mn2	133.69 (4)	C10B—C9B—H9BB	108.0
Mn3—Mn1—Mn2	60.078 (9)	C8B—C9B—H9BB	108.0
O1SA—Mn2—O1B	94.51 (6)	H9BA—C9B—H9BB	107.3
O1SA—Mn2—O1	84.57 (6)	N2B—C10B—C11B	121.1 (2)
O1B—Mn2—O1	167.16 (6)	N2B—C10B—C9B	120.51 (17)
O1SA—Mn2—N1B	168.92 (6)	C11B—C10B—C9B	118.32 (19)
O1B—Mn2—N1B	89.61 (6)	C12B—C11B—C10B	120.3 (2)
O1—Mn2—N1B	93.66 (6)	C12B—C11B—H11B	119.8
O1SA—Mn2—O2SA	99.00 (6)	C10B—C11B—H11B	119.8
O1B—Mn2—O2SA	90.61 (6)	C11B—C12B—C13B	118.4 (2)
O1—Mn2—O2SA	76.92 (5)	C11B—C12B—H12C	120.8
N1B—Mn2—O2SA	91.23 (6)	C13B—C12B—H12C	120.8
O1SA—Mn2—N2B	91.23 (6)	C14B—C13B—C12B	118.3 (2)
O1B—Mn2—N2B	97.21 (6)	C14B—C13B—H13B	120.8
O1—Mn2—N2B	95.61 (5)	C12B—C13B—H13B	120.8
N1B—Mn2—N2B	78.03 (6)	N2B—C14B—C13B	123.9 (2)
O2SA—Mn2—N2B	166.61 (6)	N2B—C14B—H14A	118.1
O1SA—Mn2—Mn3	94.44 (4)	C13B—C14B—H14A	118.1
O1B—Mn2—Mn3	128.92 (5)	C1C—O1C—Mn3	125.26 (11)
O1—Mn2—Mn3	38.72 (4)	C7C—N1C—C8C	117.21 (16)
N1B—Mn2—Mn3	90.93 (5)	C7C—N1C—Mn3	125.23 (12)
O2SA—Mn2—Mn3	38.30 (4)	C8C—N1C—Mn3	116.90 (12)
N2B—Mn2—Mn3	132.71 (4)	C14C—N2C—C10C	118.06 (18)
O1SA—Mn2—Mn1	46.05 (4)	C14C—N2C—Mn3	112.58 (14)
O1B—Mn2—Mn1	139.25 (5)	C10C—N2C—Mn3	129.29 (13)
O1—Mn2—Mn1	38.54 (4)	O1C—C1C—C2C	118.95 (17)
N1B—Mn2—Mn1	131.09 (5)	O1C—C1C—C6C	123.37 (16)
O2SA—Mn2—Mn1	87.08 (4)	C2C—C1C—C6C	117.65 (17)
N2B—Mn2—Mn1	93.94 (4)	C3C—C2C—C1C	121.53 (18)
Mn3—Mn2—Mn1	59.592 (9)	C3C—C2C—H2CA	119.2
O1C—Mn3—O2SA	93.83 (6)	C1C—C2C—H2CA	119.2
O1C—Mn3—O1	168.22 (6)	C2C—C3C—C4C	119.76 (18)
O2SA—Mn3—O1	84.73 (6)	C2C—C3C—H3CA	120.1
O1C—Mn3—N1C	89.85 (6)	C4C—C3C—H3CA	120.1
O2SA—Mn3—N1C	168.84 (6)	C5C—C4C—C3C	120.70 (19)
O1—Mn3—N1C	93.77 (6)	C5C—C4C—Cl1C	118.87 (16)
O1C—Mn3—O3SA	91.10 (6)	C3C—C4C—Cl1C	120.43 (15)
O2SA—Mn3—O3SA	97.04 (6)	C4C—C5C—C6C	119.93 (18)
O1—Mn3—O3SA	77.51 (5)	C4C—C5C—H5CA	120.0
N1C—Mn3—O3SA	93.42 (6)	C6C—C5C—H5CA	120.0
O1C—Mn3—N2C	94.14 (6)	C5C—C6C—C1C	120.36 (16)
O2SA—Mn3—N2C	91.35 (6)	C5C—C6C—C7C	117.53 (17)
O1—Mn3—N2C	97.58 (5)	C1C—C6C—C7C	121.94 (16)
N1C—Mn3—N2C	77.87 (6)	N1C—C7C—C6C	124.27 (17)
O3SA—Mn3—N2C	169.80 (5)	N1C—C7C—H7CA	117.9
O1C—Mn3—Mn1	129.62 (5)	C6C—C7C—H7CA	117.9
O2SA—Mn3—Mn1	93.74 (5)	N1C—C8C—C9C	109.69 (15)
O1—Mn3—Mn1	39.12 (4)	N1C—C8C—H8CA	109.7
N1C—Mn3—Mn1	92.01 (4)	C9C—C8C—H8CA	109.7
O3SA—Mn3—Mn1	38.53 (3)	N1C—C8C—H8CB	109.7
N2C—Mn3—Mn1	135.34 (4)	C9C—C8C—H8CB	109.7
O1C—Mn3—Mn2	139.48 (4)	H8CA—C8C—H8CB	108.2
O2SA—Mn3—Mn2	46.32 (4)	C10C—C9C—C8C	117.36 (17)
O1—Mn3—Mn2	38.52 (4)	C10C—C9C—H9CA	108.0
N1C—Mn3—Mn2	130.66 (4)	C8C—C9C—H9CA	108.0
O3SA—Mn3—Mn2	87.80 (4)	C10C—C9C—H9CB	108.0
N2C—Mn3—Mn2	93.86 (4)	C8C—C9C—H9CB	108.0
Mn1—Mn3—Mn2	60.330 (9)	H9CA—C9C—H9CB	107.2
Mn2—O1—Mn1	103.12 (6)	N2C—C10C—C11C	121.4 (2)
Mn2—O1—Mn3	102.75 (6)	N2C—C10C—C9C	120.81 (17)
Mn1—O1—Mn3	101.75 (6)	C11C—C10C—C9C	117.7 (2)
C1SA—O1SA—Mn2	126.08 (13)	C12C—C11C—C10C	119.9 (2)
C1SA—O1SA—Mn1	132.90 (13)	C12C—C11C—H11C	120.0
Mn2—O1SA—Mn1	95.74 (6)	C10C—C11C—H11C	120.0
O1SA—C1SA—H1SA	109.5	C11C—C12C—C13C	118.8 (2)
O1SA—C1SA—H1SB	109.5	C11C—C12C—H12B	120.6
H1SA—C1SA—H1SB	109.5	C13C—C12C—H12B	120.6
O1SA—C1SA—H1SC	109.5	C14C—C13C—C12C	118.1 (2)
H1SA—C1SA—H1SC	109.5	C14C—C13C—H13C	120.9
H1SB—C1SA—H1SC	109.5	C12C—C13C—H13C	120.9
C2SA—O2SA—Mn3	127.25 (12)	N2C—C14C—C13C	123.6 (2)
C2SA—O2SA—Mn2	131.89 (13)	N2C—C14C—H14C	118.2
Mn3—O2SA—Mn2	95.38 (5)	C13C—C14C—H14C	118.2
O2SA—C2SA—H2SA	109.5	O14—Cl1—O12	109.50 (14)
O2SA—C2SA—H2SB	109.5	O14—Cl1—O11	110.07 (14)
H2SA—C2SA—H2SB	109.5	O12—Cl1—O11	108.90 (12)
O2SA—C2SA—H2SC	109.5	O14—Cl1—O13	109.35 (13)
H2SA—C2SA—H2SC	109.5	O12—Cl1—O13	109.75 (13)
H2SB—C2SA—H2SC	109.5	O11—Cl1—O13	109.27 (14)
C3SA—O3SA—Mn1	123.27 (13)	C3S—O1S—C2S	111.9 (5)
C3SA—O3SA—Mn3	123.15 (12)	C2S—C1S—H1S1	109.5
Mn1—O3SA—Mn3	94.33 (5)	C2S—C1S—H1S2	109.5
O3SA—C3SA—H3SA	109.5	H1S1—C1S—H1S2	109.5
O3SA—C3SA—H3SB	109.5	C2S—C1S—H1S3	109.5
H3SA—C3SA—H3SB	109.5	H1S1—C1S—H1S3	109.5
O3SA—C3SA—H3SC	109.5	H1S2—C1S—H1S3	109.5
H3SA—C3SA—H3SC	109.5	C1S—C2S—O1S	110.7 (5)
H3SB—C3SA—H3SC	109.5	C1S—C2S—H2S1	109.5
C1A—O1A—Mn1	125.06 (12)	O1S—C2S—H2S1	109.5
C7A—N1A—C8A	119.33 (16)	C1S—C2S—H2S2	109.5
C7A—N1A—Mn1	124.48 (14)	O1S—C2S—H2S2	109.5
C8A—N1A—Mn1	116.07 (12)	H2S1—C2S—H2S2	108.1
C10A—N2A—C14A	117.84 (17)	O1S—C3S—C4S	109.1 (6)
C10A—N2A—Mn1	129.20 (13)	O1S—C3S—H3S1	109.9
C14A—N2A—Mn1	112.95 (12)	C4S—C3S—H3S1	109.9
O1A—C1A—C2A	118.92 (17)	O1S—C3S—H3S2	109.9
O1A—C1A—C6A	122.97 (18)	C4S—C3S—H3S2	109.9
C2A—C1A—C6A	118.07 (18)	H3S1—C3S—H3S2	108.3
C3A—C2A—C1A	121.22 (19)	C3S—C4S—H4S1	109.5
C3A—C2A—H2AA	119.4	C3S—C4S—H4S2	109.5
C1A—C2A—H2AA	119.4	H4S1—C4S—H4S2	109.5
C2A—C3A—C4A	119.8 (2)	C3S—C4S—H4S3	109.5
C2A—C3A—H3AA	120.1	H4S1—C4S—H4S3	109.5
C4A—C3A—H3AA	120.1	H4S2—C4S—H4S3	109.5
C5A—C4A—C3A	121.1 (2)	C3SB—O1SB—C2SB	113.7 (17)
C5A—C4A—Cl1A	119.65 (17)	C2SB—C1SB—H1S4	109.5
C3A—C4A—Cl1A	119.26 (18)	C2SB—C1SB—H1S5	109.5
C4A—C5A—C6A	120.1 (2)	H1S4—C1SB—H1S5	109.5
C4A—C5A—H5AA	119.9	C2SB—C1SB—H1S6	109.5
C6A—C5A—H5AA	119.9	H1S4—C1SB—H1S6	109.5
C5A—C6A—C1A	119.73 (19)	H1S5—C1SB—H1S6	109.5
C5A—C6A—C7A	119.00 (18)	C1SB—C2SB—O1SB	120.9 (19)
C1A—C6A—C7A	121.18 (18)	C1SB—C2SB—H2S3	107.1
N1A—C7A—C6A	125.11 (17)	O1SB—C2SB—H2S3	107.1
N1A—C7A—H7AA	117.4	C1SB—C2SB—H2S4	107.1
C6A—C7A—H7AA	117.4	O1SB—C2SB—H2S4	107.1
N1A—C8A—C9A	111.32 (15)	H2S3—C2SB—H2S4	106.8
N1A—C8A—H8AA	109.4	O1SB—C3SB—C4SB	105.4 (18)
C9A—C8A—H8AA	109.4	O1SB—C3SB—H3S3	110.7
N1A—C8A—H8AB	109.4	C4SB—C3SB—H3S3	110.7
C9A—C8A—H8AB	109.4	O1SB—C3SB—H3S4	110.7
H8AA—C8A—H8AB	108.0	C4SB—C3SB—H3S4	110.7
C10A—C9A—C8A	118.38 (16)	H3S3—C3SB—H3S4	108.8
C10A—C9A—H9AA	107.7	C3SB—C4SB—H4S4	109.5
C8A—C9A—H9AA	107.7	C3SB—C4SB—H4S5	109.5
C10A—C9A—H9AB	107.7	H4S4—C4SB—H4S5	109.5
C8A—C9A—H9AB	107.7	C3SB—C4SB—H4S6	109.5
H9AA—C9A—H9AB	107.1	H4S4—C4SB—H4S6	109.5
N2A—C10A—C11A	121.50 (19)	H4S5—C4SB—H4S6	109.5
N2A—C10A—C9A	120.97 (18)	Cl8S—C1DD—Cl7S	111.6 (14)
C11A—C10A—C9A	117.52 (18)	Cl8S—C1DD—H1D7	109.3
C12A—C11A—C10A	120.2 (2)	Cl7S—C1DD—H1D7	109.3
C12A—C11A—H11A	119.9	Cl8S—C1DD—H1D8	109.3
C10A—C11A—H11A	119.9	Cl7S—C1DD—H1D8	109.3
C11A—C12A—C13A	118.5 (2)	H1D7—C1DD—H1D8	108.0
C11A—C12A—H12A	120.7	Cl2S—C1DA—Cl1S	112.1 (8)
C13A—C12A—H12A	120.7	Cl2S—C1DA—H1D1	109.2
C14A—C13A—C12A	118.3 (2)	Cl1S—C1DA—H1D1	109.2
C14A—C13A—H13A	120.8	Cl2S—C1DA—H1D2	109.2
C12A—C13A—H13A	120.8	Cl1S—C1DA—H1D2	109.2
N2A—C14A—C13A	123.58 (19)	H1D1—C1DA—H1D2	107.9
N2A—C14A—H14B	118.2	Cl3S—C1DB—Cl4S	101.3 (12)
C13A—C14A—H14B	118.2	Cl3S—C1DB—H1D3	111.5
C1B—O1B—Mn2	123.98 (13)	Cl4S—C1DB—H1D3	111.5
C7B—N1B—C8B	117.97 (16)	Cl3S—C1DB—H1D4	111.5
C7B—N1B—Mn2	124.50 (14)	Cl4S—C1DB—H1D4	111.5
C8B—N1B—Mn2	117.28 (12)	H1D3—C1DB—H1D4	109.3
C10B—N2B—C14B	117.79 (18)	Cl6S—C1DC—Cl5S	115.1 (6)
C10B—N2B—Mn2	128.74 (14)	Cl6S—C1DC—H1D5	108.5
C14B—N2B—Mn2	113.27 (13)	Cl5S—C1DC—H1D5	108.5
O1B—C1B—C2B	118.53 (19)	Cl6S—C1DC—H1D6	108.5
O1B—C1B—C6B	123.58 (17)	Cl5S—C1DC—H1D6	108.5
C2B—C1B—C6B	117.86 (18)	H1D5—C1DC—H1D6	107.5
C3B—C2B—C1B	121.1 (2)		
			
O1B—Mn2—O1SA—C1SA	−11.50 (18)	Mn2—O1B—C1B—C2B	−149.28 (14)
O1—Mn2—O1SA—C1SA	155.65 (17)	Mn2—O1B—C1B—C6B	32.5 (2)
N1B—Mn2—O1SA—C1SA	−123.1 (3)	O1B—C1B—C2B—C3B	−177.07 (18)
O2SA—Mn2—O1SA—C1SA	79.84 (17)	C6B—C1B—C2B—C3B	1.2 (3)
N2B—Mn2—O1SA—C1SA	−108.83 (17)	C1B—C2B—C3B—C4B	−0.7 (3)
Mn3—Mn2—O1SA—C1SA	118.16 (17)	C2B—C3B—C4B—C5B	0.3 (3)
Mn1—Mn2—O1SA—C1SA	156.89 (19)	C2B—C3B—C4B—Cl1B	179.10 (17)
O1B—Mn2—O1SA—Mn1	−168.38 (6)	C3B—C4B—C5B—C6B	−0.6 (3)
O1—Mn2—O1SA—Mn1	−1.24 (5)	Cl1B—C4B—C5B—C6B	−179.33 (16)
N1B—Mn2—O1SA—Mn1	80.1 (3)	C4B—C5B—C6B—C1B	1.1 (3)
O2SA—Mn2—O1SA—Mn1	−77.04 (6)	C4B—C5B—C6B—C7B	177.37 (19)
N2B—Mn2—O1SA—Mn1	94.28 (6)	O1B—C1B—C6B—C5B	176.75 (18)
Mn3—Mn2—O1SA—Mn1	−38.73 (4)	C2B—C1B—C6B—C5B	−1.5 (3)
O1C—Mn3—O2SA—C2SA	−15.62 (18)	O1B—C1B—C6B—C7B	0.7 (3)
O1—Mn3—O2SA—C2SA	152.65 (18)	C2B—C1B—C6B—C7B	−177.51 (18)
N1C—Mn3—O2SA—C2SA	−124.6 (3)	C8B—N1B—C7B—C6B	173.93 (18)
O3SA—Mn3—O2SA—C2SA	75.96 (17)	Mn2—N1B—C7B—C6B	−0.1 (3)
N2C—Mn3—O2SA—C2SA	−109.86 (17)	C5B—C6B—C7B—N1B	166.57 (19)
Mn1—Mn3—O2SA—C2SA	114.53 (17)	C1B—C6B—C7B—N1B	−17.3 (3)
Mn2—Mn3—O2SA—C2SA	156.1 (2)	C7B—N1B—C8B—C9B	−87.5 (2)
O1C—Mn3—O2SA—Mn2	−171.71 (6)	Mn2—N1B—C8B—C9B	86.97 (16)
O1—Mn3—O2SA—Mn2	−3.44 (5)	N1B—C8B—C9B—C10B	−60.5 (2)
N1C—Mn3—O2SA—Mn2	79.3 (3)	C14B—N2B—C10B—C11B	−3.5 (3)
O3SA—Mn3—O2SA—Mn2	−80.13 (5)	Mn2—N2B—C10B—C11B	171.07 (16)
N2C—Mn3—O2SA—Mn2	94.05 (6)	C14B—N2B—C10B—C9B	174.60 (18)
Mn1—Mn3—O2SA—Mn2	−41.55 (4)	Mn2—N2B—C10B—C9B	−10.8 (3)
O1A—Mn1—O3SA—C3SA	−38.94 (16)	C8B—C9B—C10B—N2B	24.6 (3)
O1—Mn1—O3SA—C3SA	130.79 (15)	C8B—C9B—C10B—C11B	−157.28 (19)
N1A—Mn1—O3SA—C3SA	−137.6 (3)	N2B—C10B—C11B—C12B	2.0 (3)
O1SA—Mn1—O3SA—C3SA	55.09 (15)	C9B—C10B—C11B—C12B	−176.2 (2)
N2A—Mn1—O3SA—C3SA	−132.88 (15)	C10B—C11B—C12B—C13B	1.2 (4)
Mn3—Mn1—O3SA—C3SA	134.88 (17)	C11B—C12B—C13B—C14B	−2.8 (4)
Mn2—Mn1—O3SA—C3SA	93.24 (15)	C10B—N2B—C14B—C13B	1.9 (3)
O1A—Mn1—O3SA—Mn3	−173.82 (6)	Mn2—N2B—C14B—C13B	−173.49 (19)
O1—Mn1—O3SA—Mn3	−4.09 (5)	C12B—C13B—C14B—N2B	1.3 (4)
N1A—Mn1—O3SA—Mn3	87.5 (3)	O2SA—Mn3—O1C—C1C	156.03 (16)
O1SA—Mn1—O3SA—Mn3	−79.79 (6)	O1—Mn3—O1C—C1C	73.5 (3)
N2A—Mn1—O3SA—Mn3	92.24 (6)	N1C—Mn3—O1C—C1C	−34.51 (16)
Mn2—Mn1—O3SA—Mn3	−41.64 (4)	O3SA—Mn3—O1C—C1C	58.90 (16)
Mn1—O1A—C1A—C2A	−150.67 (14)	N2C—Mn3—O1C—C1C	−112.34 (16)
Mn1—O1A—C1A—C6A	31.5 (3)	Mn1—Mn3—O1C—C1C	57.96 (17)
O1A—C1A—C2A—C3A	−179.92 (19)	Mn2—Mn3—O1C—C1C	146.80 (13)
C6A—C1A—C2A—C3A	−2.0 (3)	Mn3—O1C—C1C—C2C	−151.70 (15)
C1A—C2A—C3A—C4A	1.4 (3)	Mn3—O1C—C1C—C6C	30.7 (3)
C2A—C3A—C4A—C5A	0.2 (3)	O1C—C1C—C2C—C3C	−176.40 (19)
C2A—C3A—C4A—Cl1A	178.09 (17)	C6C—C1C—C2C—C3C	1.4 (3)
C3A—C4A—C5A—C6A	−1.2 (3)	C1C—C2C—C3C—C4C	0.6 (3)
Cl1A—C4A—C5A—C6A	−179.03 (17)	C2C—C3C—C4C—C5C	−1.1 (3)
C4A—C5A—C6A—C1A	0.5 (3)	C2C—C3C—C4C—Cl1C	178.87 (17)
C4A—C5A—C6A—C7A	176.9 (2)	C3C—C4C—C5C—C6C	−0.3 (3)
O1A—C1A—C6A—C5A	178.87 (19)	Cl1C—C4C—C5C—C6C	179.73 (16)
C2A—C1A—C6A—C5A	1.0 (3)	C4C—C5C—C6C—C1C	2.2 (3)
O1A—C1A—C6A—C7A	2.6 (3)	C4C—C5C—C6C—C7C	177.53 (19)
C2A—C1A—C6A—C7A	−175.30 (18)	O1C—C1C—C6C—C5C	174.91 (19)
C8A—N1A—C7A—C6A	172.91 (18)	C2C—C1C—C6C—C5C	−2.8 (3)
Mn1—N1A—C7A—C6A	−3.0 (3)	O1C—C1C—C6C—C7C	−0.2 (3)
C5A—C6A—C7A—N1A	166.8 (2)	C2C—C1C—C6C—C7C	−177.84 (19)
C1A—C6A—C7A—N1A	−16.9 (3)	C8C—N1C—C7C—C6C	170.75 (17)
C7A—N1A—C8A—C9A	−91.0 (2)	Mn3—N1C—C7C—C6C	0.3 (3)
Mn1—N1A—C8A—C9A	85.20 (17)	C5C—C6C—C7C—N1C	169.30 (19)
N1A—C8A—C9A—C10A	−52.4 (2)	C1C—C6C—C7C—N1C	−15.5 (3)
C14A—N2A—C10A—C11A	−2.5 (3)	C7C—N1C—C8C—C9C	−82.6 (2)
Mn1—N2A—C10A—C11A	176.53 (14)	Mn3—N1C—C8C—C9C	88.59 (17)
C14A—N2A—C10A—C9A	176.13 (18)	N1C—C8C—C9C—C10C	−56.7 (2)
Mn1—N2A—C10A—C9A	−4.8 (3)	C14C—N2C—C10C—C11C	−2.3 (3)
C8A—C9A—C10A—N2A	14.3 (3)	Mn3—N2C—C10C—C11C	−179.10 (15)
C8A—C9A—C10A—C11A	−167.00 (18)	C14C—N2C—C10C—C9C	174.83 (18)
N2A—C10A—C11A—C12A	1.4 (3)	Mn3—N2C—C10C—C9C	−2.0 (3)
C9A—C10A—C11A—C12A	−177.28 (19)	C8C—C9C—C10C—N2C	16.5 (3)
C10A—C11A—C12A—C13A	0.7 (3)	C8C—C9C—C10C—C11C	−166.31 (18)
C11A—C12A—C13A—C14A	−1.5 (3)	N2C—C10C—C11C—C12C	1.4 (3)
C10A—N2A—C14A—C13A	1.6 (3)	C9C—C10C—C11C—C12C	−175.8 (2)
Mn1—N2A—C14A—C13A	−177.56 (16)	C10C—C11C—C12C—C13C	0.6 (3)
C12A—C13A—C14A—N2A	0.4 (3)	C11C—C12C—C13C—C14C	−1.7 (3)
O1SA—Mn2—O1B—C1B	152.89 (14)	C10C—N2C—C14C—C13C	1.2 (3)
O1—Mn2—O1B—C1B	67.5 (3)	Mn3—N2C—C14C—C13C	178.53 (17)
N1B—Mn2—O1B—C1B	−37.41 (14)	C12C—C13C—C14C—N2C	0.8 (3)
O2SA—Mn2—O1B—C1B	53.82 (14)	C3S—O1S—C2S—C1S	176.8 (5)
N2B—Mn2—O1B—C1B	−115.29 (14)	C2S—O1S—C3S—C4S	−177.0 (5)
Mn3—Mn2—O1B—C1B	53.48 (16)	C3SB—O1SB—C2SB—C1SB	12 (3)
Mn1—Mn2—O1B—C1B	140.06 (12)	C2SB—O1SB—C3SB—C4SB	179 (2)

### Hydrogen-bond geometry (Å, º)

**Table:**

*D*—H···*A*	*D*—H	H···*A*	*D*···*A*	*D*—H···*A*
C2*SA*—H2*SA*···O1*C*	0.98	2.42	2.991 (3)	117
C5*A*—H5*AA*···Cl8*S*	0.95	2.94	3.874 (11)	168
C5*A*—H5*AA*···Cl3*S*^i^	0.95	2.72	3.39 (2)	128
C8*A*—H8*AA*···O1	0.99	2.40	3.061 (2)	123
C14*A*—H14*B*···O3*SA*	0.95	2.44	3.036 (2)	120
C3*B*—H3*BA*···Cl1*C*^ii^	0.95	2.91	3.758 (2)	149
C8*B*—H8*BA*···O1	0.99	2.45	3.089 (2)	122
C9*B*—H9*BA*···O1*A*^iii^	0.99	2.47	3.335 (2)	145
C9*B*—H9*BB*···O11^iv^	0.99	2.54	3.241 (3)	128
C14*B*—H14*A*···O1*SA*	0.95	2.53	3.116 (2)	120
C5*C*—H5*CA*···O14	0.95	2.38	3.300 (3)	164
C7*C*—H7*CA*···O13	0.95	2.55	3.478 (3)	164
C8*C*—H8*CA*···O1	0.99	2.43	3.079 (2)	123
C9*C*—H9*CA*···Cl7*S*^v^	0.99	2.68	3.631 (11)	161
C9*C*—H9*CB*···O12^iv^	0.99	2.57	3.371 (3)	138
C9*C*—H9*CB*···O13^iv^	0.99	2.64	3.403 (3)	134
C13*C*—H13*C*···Cl1*S*^vi^	0.95	2.80	3.698 (6)	159
C13*C*—H13*C*···Cl4*S*^vi^	0.95	2.52	3.441 (12)	163
C13*C*—H13*C*···Cl5*S*^vi^	0.95	2.91	3.843 (5)	169
C14*C*—H14*C*···O2*SA*	0.95	2.47	3.082 (3)	122
C1*DA*—H1*D*1···O11	0.99	2.53	3.201 (15)	125
C1*DA*—H1*D*1···O14	0.99	2.65	3.63 (2)	169
C1*DC*—H1*D*6···O11	0.99	2.24	3.176 (10)	158

Symmetry codes: (i) *x*−1, *y*, *z*; (ii) −*x*+1, −*y*+1, −*z*+1; (iii) −*x*+1/2, *y*−1/2, −*z*+3/2; (iv) −*x*+1, −*y*+1, −*z*+2; (v) *x*+1, *y*, *z*; (vi) −*x*+3/2, *y*−1/2, −*z*+3/2.
